# Improving Identification of Tic Disorders in Children

**DOI:** 10.1080/23794925.2024.2324775

**Published:** 2024-03-21

**Authors:** Rebecca C. Wardrop, Adam B. Lewin, Heather R. Adams, Jennifer A. Vermilion, Steven P. Cuffe, Melissa L. Danielson, Rebecca H. Bitsko, Bo Cai, James W. Hardin

**Affiliations:** a Department of Epidemiology and Biostatistics, University of South Carolina, Columbia, USA; b Department of Pediatrics, University of South Florida, Tampa, USA; c Department of Neurology & Department of Pediatrics, University of Rochester Medical Center, Rochester, New York, USA; d Department of Psychiatry, University of Florida College of Medicine – Jacksonville, USA; e Division of Human Development and Disability, Centers for Disease Control and Prevention, Atlanta, Georgia, USA

## Abstract

This study combines data from five studies in a quantitative modeling approach to improve identification of tics and tic disorders using two questionnaires (the Motor or Vocal Inventory of Tics and the Description of Tic Symptoms), administered to parents and children $\left(N=1,307\right)$. Combining final diagnoses (positive or negative for tic disorder) with data from recently developed questionnaires implemented to assist in the identification of tics and tic disorders in children, we investigate methods for predicting positive diagnosis while also identifying which items in the questionnaires are most predictive. Logistic regression and random forest models are compared using various summary statistics. We further discuss the differences in errors (false positives versus false negatives) in the specification of predictive model tuning parameters. Compared to logistic regression models, random forest models provided comparable and often superior predictive abilities and were also more useful in summarizing the contributions to predictions from individual questions. The combined analyses identified a subset of screener questions that were the best predictors of tic disorders; the identified questions differed based on parent or self-report. These results provide information to inform the future development of tools to screen for tics in a variety of healthcare and epidemiological settings.

## Introduction

Tics are involuntary, recurring movements or sounds that may interfere with typical behavior (American Psychiatric Association, 2013). Many individuals with tic disorders describe an accompanying premonitory sensation or urge to perform the tic, with improved self-awareness of premonitory tic experiences developing by about age 10–12 years old (Woods et al., 2005). Up to 20% of children may have tics at some point during their development, though the majority will not go on to develop a persistent tic disorder (Kurlan et al., 2001). Based on criteria in the Diagnostic and Statistical Manual of Mental Disorders, 5^th^ edition (DSM-5), the presence of at least one tic prior to the age of 18 that is not caused by a medication or other medical condition and with the tic(s) lasting for less than one year meets criteria for a provisional tic disorder, while persistent tic disorders, including Tourette syndrome (also referred to as Tourette’s disorder), require that tics persist for at least one year (American Psychiatric Association, 2013).

While there are numerous measures of tic severity there are fewer measures for screening and diagnosis of tic disorders (Gaffney et al., 1994; Leckman et al., 1989; Linazasoro et al., 2006; Martino et al., 2017; Shytle et al., 2003). In addition to having different purposes (e.g., screening vs. severity), measures also differ by informant (e.g., parent, self, teacher, clinical assessment). Large epidemiologic studies often rely on parent-, self-, or teacher report rather than using clinical assessment which requires additional time and resources (e.g., Costello et al., 1996; Danielson et al., 2021; Kessler et al., 2009). Previous studies of measures to identify tics have shown differences in reporting by informant type (Adams et al., 2023; Cubo et al., 2011).

Two measures that were developed recently for use in the identification of tics and tic disorders in epidemiologic studies, and include both parent- and self-report questionnaires, are the 10-item Motor or Vocal Inventory of Tics (MOVeIT-10) screener and the Description of Tic Symptoms (DoTS), a diagnostic measure (Adams et al., 2023; Lewin et al., 2024). The MOVeIT-10 is a screening tool that assesses for the presence/absence of tics. The DoTS is a diagnostic measure, and assesses for the presence of potential tics, characteristics of tics, age of onset, and duration of tics. The objective of this project was to investigate which questions from the two instruments most strongly predict which children have a tic disorder compared with clinical assessment. Identification of the most predictive questions could be used to adapt or develop better assessments to identify tics and tic disorders in clinical and research settings. A secondary objective was to compare the psychometrics of, and results produced by, two analytic methods, logistic regression and random forests, to identify the importance of individual items.

## Materials and methods

### Participants

The data reflect information on 1,307 children aged 2–20 years collected from their parents and the children themselves if they were age 8 years or older, across five samples from diverse communities in New York and Florida. Details on the five studies that contributed to the combined data set are summarized in Table 1 (see also Adams et al., 2023; Danielson et al., 2021; Lewin et al., 2024; Vermilion et al., 2023).

#### Project to learn about youth mental (“PLAY-MH”)

The first study included data from the Florida site of the school-based multistage study, the Project to Learn about Youth Mental Health (PLAY-MH) that collected teacher screening (stage 1) and parent diagnostic interview, including the DoTS (stage 2), data to understand community prevalence and treatment patterns for child mental disorders (Danielson et al., 2021). In Stage 1 at the Florida site, all students in nine pre-selected schools were eligible for screening which was completed by a primary teacher for elementary students, and by a teacher for a designated period for middle and high school students. Based on the screening, students were invited for Stage 2, where the DoTS was completed. For the Florida site only, children with potential tics indicated on the DoTS were invited for a stage 3 assessment for tic disorders. The gold standard assessment was the Schedule for Affective Disorder and Schizophrenia for School-Age Children-Present and Lifetime (K-SADS-PL; see additional details below) (Kaufman et al., 1997), administered by a child and adolescent psychiatrist (with experience in diagnosing tic disorders, but not a specialty tic clinic). There were 93 children aged 8–20 years enrolled in the stage 3 assessment for tic disorders.

#### University of South Florida MOVeIT development study (“MOVeIT development”)

The second study focused on the development of the MOVeIT and included the final 42 children ages 4–17 years and their caregivers participating in a screener development project (see Lewin et al., 2024). Eleven participants were recruited from normal patient flow at a specialty clinic for obsessive-compulsive disorder (OCD), anxiety, and tic disorders (although many children with tics are seen in the University of South Florida (USF) Rothman Center Clinic, all eligible participants were approached without the investigator knowledge of tic disorder presence). In addition, 31 participants were recruited from consecutive flow at the USF General Pediatric Clinic for initial testing of the MOVeIT-14 in a general/non-specialty setting. Participants were administered the Yale Global Tic Severity Scale (YGTSS; Leckman et al., 1989) and reviewed by expert clinicians to determine tic status. This project created the 14-item measure (MOVeIT-14) (Lewin et al., 2024).

#### University of Rochester validation study (“validation study”)

The third study (Adams et al., 2023) included 100 children aged 6–17 years who met criteria for a persistent tic disorder (n=57; 55 were diagnosed prior to participation in the study and 2 were identified as meeting criteria for a persistent tic disorder as part of the study) or with no known history of tics and who did not meet diagnostic criteria for a tic disorder (n=43). Participants with tic disorders were identified through a tic specialty clinic, and control participants were identified through community settings (e.g., libraries, sports clubs) and from primary care clinics. Participants and their parents were enrolled in a case-control study to evaluate sensitivity and specificity of the MOVeIT −14 and the DoTS. Analyses assessed the performance of the MOVeIT-14 and the 10 items (a subset of the MOVeIT-14) that make up the MOVeIT-10. These index tests were compared against the reference of a gold-standard evaluation by a tic expert (a clinician with expertise in diagnosis and treatment of tic disorders). The order in which participants completed these questionnaires was assigned randomly to minimize potential order effects or bias related to systematically completing one form or the other first.

#### University of Rochester developmental-behavioral pediatrics (“DBP”)

The fourth study (Vermilion et al., 2023) enrolled 266 children aged 2–15 years from a Developmental-Behavioral Pediatrics (DBP) clinic to evaluate sensitivity and specificity of the MOVeIT-14 in a sample of children with intellectual and developmental disability (IDD). Here, the major aim was to determine if the MOVeIT-14, or the 10 items included in the MOVeIT-10 could identify tics in a sample enriched for stereotypy, another common movement disorder of childhood that is highly prevalent in children with IDD. Participants were recruited prior to or during regular clinic visits with invitations to participate sent by mail, through the electronic health record, by phone, and in-person. Evaluation by a tic expert was also used as the gold standard reference for this study.

#### University of South Florida Tics as a marker study (“tics as a marker”)

The fifth study included 799 children recruited from an urban, southeastern pediatric primary care clinic (USF) as part of a study to (a) evaluate performance of screening measures in a general pediatric setting, and (b) to determine if the identification of tics could be used as a “marker” for symptoms of other conditions (e.g., OCD). Children aged 4–17 years and their parents were recruited through the consecutive flow of the USF General Pediatrics Clinic. Tics were not discussed during consent/recruitment. Children/caregivers were administered a tic screening measure (either the MOVeIT-10 or the first page of the DoTS; screeners were alternated) prior to any other assessment to avoid priming the respondent’s report of tics. Following the screener, the YGTSS and K-SADS-PL tic disorder module were administered as the gold standard to determine tic status.

Institutional Review Board approval was received for each study (Florida PLAY-MH: 2014-U-1382; USF MOVeIT development: Pro00023632; UR validation study: RSRB00064456; UR DBP Project: RSRB00070865; USF Tics as a marker: Pro00037340). Informed assent/consent was obtained from all participants.

### Measures

#### Motor or vocal inventory of tics (MOVeIT)

The MOVeIT was developed as a screener to identify the presence and frequency of potential tics. Initially the MOVeIT included 14 items (MOVeIT-14) but then was shortened to 10 items (MOVeIT-10; Adams et al., 2023; Lewin et al., 2024). Each item mentions general characteristics of motor and/or vocal tics (e.g., makes the same movements over and over) and some items include specific examples of tics (e.g., constant blinking, grunts) and the respondent selects whether each happens never, sometimes, or often (see Adams et al., 2023 for all MOVeIT-10 items; Lewin et al., 2024). In the “MOVeIT development study,” both the MOVeIT-14 parent and child versions were highly correlated with the YGTSS (r=0.78 and 0.68, respectively), and the parent version correctly classified 83% of children and the child version correctly classified 62% of children (Lewin et al., 2024). In the “Validation Study,” compared to expert clinical assessment, the sensitivity of both the parent and child MOVeIT-10 was over 90% and the specificity was 100% for parent report and 89% for child report; children with tic disorders were identified through a specialty tic clinic (Adams et al., 2023). Although some of the studies included here used the MOVeIT-14, the analyses presented here focus on the 10 items of the MOVeIT-10, with scores ranging from 0 (response of “never” to all questions) to 20 (response of “often” to all questions).

#### The description of tic symptoms (DoTS)

The DoTS (see Adams et al., 2023 for full measure) is a two-page questionnaire to assess for the presence of tics as well as diagnostic criteria for tic disorders (Adams et al., 2023). The first page assesses for the presence of potential tics using questions from previously developed measures: the Motor tic, Obsessions and compulsions, Vocal tic Evaluation Survey (MOVES) (Gaffney et al., 1994; Lewin et al., 2023), the Diagnostic Interview Schedule for Children, version IV (Shaffer et al., 2000), and the Proxy Report Questionnaire (Cubo et al., 2011; Linazasoro et al., 2006). If an individual endorses any potential tic on the first page, they continue to complete the second page to report on characteristics of potential tics (e.g., ability to suppress), age of onset and duration of potential tics, and impairment associated with potential tics. Information from the first page on types of tics reported (motor or vocal) and on page 2 on age of onset and duration of tics can be used to determine if a child has potential tics and to categorize tic disorder based on DSM-5 criteria for tic disorders, including Tourette syndrome (American Psychiatric Association, 2013). Of note, the DoTS does not capture information about the remaining diagnostic criterion, namely, exclusion of children whose tic symptoms have another medical cause. The “Validation Study” that included children with tic disorders recruited through a specialty clinic found that, compared to expert clinical assessment, the parent and child DoTS both had 100% sensitivity for identifying any tic disorder; specificity was 93% for the parent DoTS and 76% for the child DoTS (Adams et al., 2023). Unpublished data suggest the MOVeIT and DoTS do not perform as well in general population setting (Bitsko et al., unpublished data from PLAY-MH; Lewin et al., unpublished data from “Tics as a Marker” study). Only responses to the first page of the DoTS were used in this study (because these items focus on tic presence specifically).

#### Definition of tic disorder

Gold standard measures differed across studies and included the K-SADS-PL tic disorder module, the YGTSS, and expert assessment to determine whether children had a tic or met criteria for a tic disorder (see Table 1). The K-SADS-PL is a semi-structured integrated parent-child interview that relies on a clinician to assess the Diagnostic and Statistical Manual of Mental Disorders, 4^th^ edition (DSM-IV) (American Psychiatric Association, 2000) diagnostic criteria for a number of mental disorders, including tic disorders, and allows for clinical judgment when integrating the data (Kaufman et al., 1997). Validation studies of translated (i.e., not English) K-SADS-PL in clinical populations in Iran, and Sweden have shown high agreement (Kappa range 0.81–0.90) between lifetime diagnoses of tic disorders on the K-SADS-PL compared with clinical diagnosis, with samples of children with tic disorders ranging from 5–20 (Ghanizadeh et al., 2006; Jarbin et al., 2017; Shahrivar et al., 2010); however, one study in Korea found a lower agreement (Kappa = 0.40) in a clinical sample that included 17 children with tic disorders (Kim et al., 2004). Children were considered to have a tic disorder if their K-SADS-PL interview indicated Tourette syndrome, persistent motor or vocal tic disorder, provisional tic disorder, or tic disorder not otherwise specified. The YGTSS is a clinician-rated tool used to assess the presence and severity of motor and vocal tics, that has been validated in a number of studies, and used widely in clinical and research settings (Leckman et al., 1989; Martino et al., 2017; McGuire et al., 2018). Based on the YGTSS, children were considered to have a tic if total severity scores were greater than 0. Expert assessment consisted of a clinical gold standard evaluation for tics and tic disorders conducted by clinical researchers well-versed in tics and other movement disorders of childhood (pediatric neurologist; pediatric neurology nurse practitioner; child neuropsychologist, pediatric neurologist, pediatric neurology nurse), and benchmarked against DSM-5 diagnostic criteria (Adams et al., 2023; American Psychiatric Association, 2013; Vermilion et al., 2023).

### Data analysis

The combined data set is composed of basic demographic information for the children (age, sex, and race/ethnicity (non-Hispanic White, non-Hispanic Black, and other or multiples races/ethnicities)) and responses to the MOVeIT-10 and DoTS questionnaires that were completed by parents and children. Of the 1,205 children with known tic disorder status, 822 had information collected from a MOVeIT questionnaire (323 completed by the child and 818 completed by the parent with 319 completed by both parent and child) and 484 had information from the DoTS questionnaire (303 completed by the child and 479 completed by the parent with 298 completed by both parent and child).

#### Data sampling

To estimate the predictive models (described below), 70% of each data set was selected to be used as a training set and the remaining 30% was set aside to be used as a validation set (Silge et al., 2022). Six non-mutually exclusive sets of data (random samples selected independently such that there can be common observations across sets) that included child demographic information were evaluated with the predictive models: MOVeIT-10 parent responses, child responses, and combined parent and child responses; DoTS parent responses, child responses, and combined parent and child responses. No observations are repeated within a data set, but they may be included in more than one set. Each data set was evaluated using a logistic regression model and a random forest model.

#### Logistic regression

We used logistic regression to model the probability, p, of a particular child having tic disorder as a function of questionnaire items and demographic characteristics (age, sex, race/ethnicity). Thus, the probability of tic disorder was modeled as

$$p=\frac{1}{1\;+\;\exp\left[-\left(\beta_{0}+\Sigma_{j=1}^{m}\beta_{j}x_{j}\right)\right]}$$

where ${\text{β}}_{0}$ is the intercept, m is the number of questionnaire items and demographic variables, ${\text{β}}_{\text{j}}$ are the derived coefficients for the m questions/demographics, and $\chi_{j}$ are the answers to the questions (demographics and individual questions from the questionnaires). When presented with a new set of answers to a MOVeIT-10 or DoTS questionnaire, the estimated model (the values of the intercept and coefficients) defines the probability that a child has a tic disorder given their demographics and questionnaire answers.

A cutoff was chosen such that any predicted probability above the cutoff was assumed to be a positive prediction (predicted tic disorder) and any prediction below the cutoff was assumed to be a negative prediction (predicted to not have tic disorder). A comparison of the predicted outcome versus the known outcome illustrates four possible outcomes for each predicted probability under a specific cutoff: (1) correctly predict tic disorder (true positive), (2) erroneously predict tic disorder (false positive), (3) erroneously predict no tic disorder (false negative), and (4) correctly predict no tic disorder (true negative).

#### Random forest

Random forest is an ensemble machine learning algorithm introduced by Breiman and Cutler (Breiman, 2001; Cutler et al., 2012) to combine multiple weak classification and regression trees which perform slightly better than random guessing to build a stronger model with arbitrarily better prediction accuracy. A defining feature of random forest models is boosted aggregation (bagging) (Breiman, 1996) which involves taking new bootstrap samples to build each classification tree. This results in approximately one-third of the data not being included in the sample used to build an individual tree. Such data are considered out-of-bag (OOB) and are used to estimate the generalization error and other evaluation metrics of the final predictive model such as sensitivity and specificity.

Ten-fold cross validation is used to tune the number of predictors randomly selected at each node to determine the best split. In this approach, the data are randomly separated into 10 groups. At iteration 1, a training dataset (suitable for model estimation) is defined as group 1 through group 9 leaving group 10 as a test dataset (suitable for evaluating the model defined by the other groups). Leaving out each group as the training set, there are 10 such pairs of training and testing datasets. Results from the collection are then averaged.

The final model is selected to maximize the OOB estimated AUC (area under the curve plotting the false positive rate vs. the true positive rate). The proportion of trees in the random forest model that classify a child as having a tic disorder based on their answers to the questionnaires represents the random forest prediction of whether a child has a tic disorder.

#### Statistical analysis

Statistical analyses were conducted in R (Kuhn, 2022; Liaw & Wiener, 2002; R Core Team, 2021). Weighted Youden’s indices were used to select the cut points to determine which predicted probabilities in logistic regression and which proportion of trees in the random forest would indicate a tic disorder. Weighted indices were used to emphasize sensitivity over specificity to decrease the likelihood of false negatives; a range of different weights were tested, and the final cut points were established based off of a weighted Youden’s index with a weight of 3. The performance of the models using the DoTS and MOVeIT-10 items to predict whether a child has a tic disorder were evaluated using sensitivity and specificity.

$$sensitivity=\frac{TP}{TP+FN},$$

$$specificity=\frac{TN}{TN+FP}$$

where TP TP, TN, FP, and FN are the number of true positives, true negatives, false positives, and false negatives, respectively. Sensitivity was prioritized to evaluate instrument performance based on the identified cut points because the goal was to identify children who require further evaluation for tic disorders; therefore, avoiding false negatives is more important than incurring false positives.

A Wald test of the significance of each individual coefficient in the logistic regression models was used to assess whether a predictor has a significant relationship with the outcome at a significance level of α=0.10; this significance level was chosen to be less conservative than the traditional value of 0.05 as the goal was to identify possible items associated with the outcome. In the random forest models, variable importance is measured by the decrease in accuracy when the variable’s OOB values are permuted. Thus, a negative value for importance would indicate increased accuracy when the variable was excluded from the random forest – it does not imply an inverse relationship between the associated question and tic disorder diagnosis. Variable importance was assessed at a cutoff value of 0.5; the cutoff applied in defining a random forest affects the number of possible trees. A sensitivity analysis indicated no appreciable difference in our reported results when the cutoff was between 0.4 and 0.6. These results will identify the items in each questionnaire that possess the most predictive power in determining a child’s tic disorder status.

The random forest approach produces a much more accurate prediction than does a single logistic regression model (Couronné et al., 2018). In fact, the random forest prediction algorithm owes its popularity to the fact that random forest models favor prediction over explanation (Breiman, 2001; Couronné et al., 2018). Logistic regression calculates parameters that estimate the average predicted probability for each covariate pattern (set of values for the questionnaire items and demographics). However, random forest models attempt to build decision trees that maximize the agreement of prediction and observation for each covariate pattern. Thus, logistic regression can identify how specific questions might change the average predicted probability while random forests make no attempt to illuminate associations.

#### Sensitivity analysis

Sensitivity analyses were conducted excluding data collected from children recruited at tic disorder specialty clinics and developmental and behavioral pediatric clinics to determine how the models performed when only including children recruited from general population settings. This excluded data from the USF MOVeIT development study, the UR Validation Study: MOVeIT & DoTS, and the UR AUCD Project MOVeIT. All logistic regression and random forest models were re-estimated based on these limited data sets and Wald tests of significance of each coefficient in the logistic regression models were conducted and variable importance measures were calculated in the random forest models.

## Results

### Population characteristics

The percentage of study participants who had a known tic disorder status and at least one response to a MOVeIT or DoTS questionnaire was 92.2% (n=1,205). Of these 1,205 children, 705 (58.5%) were male and 500 (41.5%) were female. Children ranged in age from 2 to 20 years with a median age of 9 years. There were 139 (11.5%) children under the age of 5 included in the study. There were 537 (44.6%) non-Hispanic White children, 335 (27.8%) non-Hispanic Black children, and 325 (27.0%) children were of other or multiple races/ethnicities. Children with missing information on their race/ethnicity made up 0.6% (8 children) of the study population.

### Prediction of tic disorder

Results regarding the prediction of tic disorders using MOVeIT-10 models and DoTS models are provided in Tables 2 and 3, respectively (full measures are included in Adams et al., 2023). When maximizing the weighted Youden’s index, all random forest MOVeIT-10 models had sensitivity greater than 83%, consistently out-performing the corresponding logistic regression models. The specificity in the random forest and logistic regression MOVeIT-10 models was always above 40%. Similarly, all logistic regression and random forest DoTS models achieved sensitivity greater than or equal to 85%. Specificity in all DoTS models also remained high (greater than or equal to 75%), indicating that most children without a tic disorder were also being accurately classified.

### Identification of MOVeIT-10 item importance

Table 4 includes results of the variable importance measure for all MOVeIT-10 random forest models. Estimated odds ratios in the logistic regression MOVeIT-10 models are provided in Table 5. Items included in the MOVeIT-10 questionnaire are detailed in Adams et al. (2023). Items are summarized in the text below based on whether they assessed movements and/or sounds, used the wording “over and over,” “even if I don’t want to,” or “were hard to keep from doing,” and if they included examples of possible tics.

Considering parent and child report together, MOVeIT-10 parent report on items 1 (movements/sounds over and over), 4 (movements/sounds, hard to keep from doing, with examples), 5 (movements over and over, with examples), and 6 (movements/sounds over and over, with examples) were most important in the random forest models, followed by parent report on items 7 (sounds over and over, hard to keep from doing) and 9 (sounds over and over); child report items 1 (movements/sounds over and over), 9 (sounds over and over), and 10 (movements over and over, hard to keep from doing) were most important. In the logistic regression models, only parent report on items 7 (sounds over and over, hard to keep from doing) and 9 (sounds over and over) were significant, as well as self-report items 7, 9, and 10 (movements over and over, hard to keep from doing); parent report on item 9 was only significant in the unadjusted model while the other items were significant in both the unadjusted and adjusted models). In sensitivity analyses restricting the sample to community-based studies (see Supplemental Tables S6 and S7), and considering parent and child report together, parent report items 1 (movements/sounds over and over), 4 (movements/sounds over and over, hard to keep from doing, with examples), 6 (movements/sounds over and over, with examples), and 7 (sounds over and over, hard to keep from doing) and child items 4, 5 (movements over and over, with examples), and 10 (movements over and over, hard to keep from doing) were the most important in the random forest models. For the logistic regression results, only parent report item 7 (sounds over and over, hard to keep from doing) in both models (i.e., with and without demographics) and self-report item 3 (movements even if I don’t want to, with examples) in the model adjusted for demographics were significant.

Considering parent responses alone, MOVeIT-10 parent items 4 (movements/sounds over and over, hard to keep from doing, with examples), 5 (movements over and over, with examples), 6 (movements/sounds over and over, with examples), and 10 (movements over and over, hard to keep from doing) were most important in the random forest models (regardless of accounting for demographics), and item 1 (movements/sounds over and over) was also among the most important when adjusting for demographics; only item 5 (movements over and over, with examples) was significant in the logistic regression models. In the sensitivity analysis, items 4, 5, and 6 were most important in the random forest models, and item 6 was the only significant item in the logistic regression models.

Considering child report alone, MOVeIT-10 items 1 (movements/sounds over and over), 2 (sounds, even if I don’t want to), 4 (movements/sounds over and over, hard to keep from doing, with examples, 9 (sounds over and over), and 10 (movements over and over, hard to keep from doing) were most important in the random forest models; item 6 (movements/sounds over and over, with examples) was among the most important items only when adjusting for demographics. In the logistic regression models, items 1 and 10 (item 10 was only significant after adjusting for demographics) had a significant positive association and item 8 (sounds/movements, even if I don’t want to) had a significant negative association. In the sensitivity analysis, items 2 (sounds, even if I don’t want to) and 10 (movements over and over, hard to keep from doing) were most important in the random forest models, and item 2 was also significant in the logistic regression models.

Considering demographic variables in the MOVeIT-10 models, race had the highest variable importance for the combined parent and child random forest model, while age and race were highest for the parent only model and race and sex were highest for the child only model. In the logistic regression models, females had significantly lower odds of having a tic disorder compared to males based on the combined parent and child and child report alone models. Participants of other (excluding non-Hispanic Black) races had significantly lower odds of having a tic disorder relative to non-Hispanic White participants for the parent only and child only models, and non-Hispanic Black children had lower odds for the models with combined parent and child and child only data. In the logistic model including only parent responses, age had a significant positive association with tic disorder indicating that older participants had higher odds of tic disorder.

#### Identification of DoTS item importance

Information on the identification of important items from the DoTS random forest and logistic models is provided in Tables 6 and 7, respectively. When both parent and child report were considered, parent report on DoTS items 1a (makes noises (like grunts)), 1c (same jerk or twitch), and both parent- and self-report on 4a (ever had tics) were the most important based on random forest models and were also significant in the unadjusted logistic regression models. After adjusting for demographic factors in the logistic regression models, parent-report on 1a (makes noises like grunts) and both parent- and child-report on 4a (ever had tics) remained significant; in addition, self-report on item 1b (body jerks) was significant, but with a negative association when adjusting for demographic factors. In the sensitivity analysis (restricted to community-based studies), for the combined parent and child random forest models, both parent and self-report on items 1a (makes noises like grunts), 1c (same jerk or twitch), and 4a (ever had tics) were the most important items. In the corresponding logistic regression models, parent report on 1a and 4a and self-report on items 2 (makes short movements) and 4a (ever had tics) had significant, positive associations with tic disorders (see Supplemental Tables S1 and S2).

Considering parent report alone, DoTS items 1c (same jerk or twitch), 4a (ever had tics), and 4b (currently has tics) were most important based on random forest models. Both 1c and 4a were significant in the parent only logistic regression models while 4b was not; after adjusting for demographic factors only 4a (and not 1c) was significant. Two additional items were significant in the logistic regression models based on parent report only: 1a (makes noises like grunts; significant positive association before and after adjusting for demographics) and 1e (feels pressure to talk, shout, or scream; significant negative association before and after adjusting for demographics). Based on the sensitivity analysis, items 1a and 4a were most important based on the random forest models, but only 1a (makes noises like grunts) was significant in the logistic regression models; in addition, items 1e (feels pressure to talk, shout, or scream; negative association) and 2 (makes short movements) were significant in the logistic regression models before (1e) or after (2) adjusting for demographics.

Considering child report alone, DoTS items 4a (ever had tics) and 4b (currently has tics) were most important based on random forest models but only 4a (ever) was significant (with and without adjustment for demographics) in the logistic regression models. In addition, based on child report alone, items 1c (same jerk or twitch) and 1d (can’t control all movements) were significant and positively associated with tic disorders, although the association of 1d was no longer significant after adjustment for demographic variables. Child report on items 1e (feels pressure to talk, shout, or scream) and 1f (habits or movements when nervous) was significantly negatively associated with tic disorders in the unadjusted logistic regression models. In the sensitivity analyses with community-based samples only, items 1c (same jerk or twitch) and 4a (ever tics) were most important in the random forest models, but only 4a was significant in the logistic regression models; in addition, items 1b (body jerks) and 4b (current tics) both had significant negative associations with tic disorders before (4b) or after (1b) adjustment for demographic factors. We anticipated positive associations between these items and tic disorder. The negative associations could be due to a Type I error of a null association; results from other studies could be informative on whether that is the case.

Demographic variables were also important and significant in random forest and logistic regression models for the DoTS. Both race and age had positive variable importance in the parent and child combined, parent only, and child only random forest models; sex also had positive variable importance in the child only random forest model. In the logistic regression models, children with “other/multiple” race had significantly lower odds of having a tic disorder compared to non-Hispanic White children in all three models (combined, parent-only, child only). In the child report only model, females had lower odds than males and non-Hispanic Black children had lower odds than non-Hispanic White children to have a tic disorder.

## Discussion

Early identification of tics increases the opportunity for treatment if needed which may lead to improved health and well-being. Validated measures can be incorporated into future epidemiologic and research studies to improve our understanding of the prevalence of tics and tic disorders. The results from this study provide valuable information that can be used to adjust existing measures or inform the development of new measures that are designed to screen for the presence of tics and tic disorders. Item performance varied by parent and child report, as well as for the full sample compared to when we restricted the sample to only include participants from general population settings (vs. recruited through specialty tic clinics where those with tics already had a diagnosis). These findings support testing of revised measures in general population settings, and to examine performance by different reporters.

Previous studies have shown the MOVe-IT and DoTS show promise as screening and diagnostic instruments (Adams et al., 2023), but have not performed as well in general population settings compared to studies where children with previously diagnosed tic disorders make up a substantial portion of participants (Bitsko et al., unpublished data from PLAY-MH; Lewin et al., unpublished data from tics as a marker). Specifically, the individual analyses of the unpublished DoTS data showed that only one-third of students who met criteria for a persistent tic disorder on the DoTS were also determined to have a tic disorder based on clinical assessment (Bitsko et al., unpublished data from PLAY-MH). Importantly, data from these three previous studies (Adams et al., 2023; Bitsko et al., unpublished data from PLAY-MH; Lewin et al., unpublished data from tics as a marker) are included in the analyses presented here. Together, these results suggest improvements can be made to both measures.

Based on the results reported here, DoTS items 2 and 3, which require write-in responses of potential tics, can be eliminated from future measures. In addition to not performing well in either random forest or logistic regression analyses, coding of these items requires tic expertise, and experts found categorization of many of the write-in responses challenging without additional information (Bitsko et al., unpublished data from PLAY-MH). DoTS items 1a (makes noises like grunts) and 1c (same jerk or twitch) were items that provided predictive value and could all be considered for future measures on tics and tic disorders, as the associations of either or both parent and child responses were significant in at least one logistic regression model and had positive and often high variable importance in random forest models. Both parent and child responses to DoTS item 4a (ever had tics) were consistently among the most important if not the most important item in all random forest models and was significant in every logistic regression model. This item also remained significant and important in sensitivity analyses. Thus, item 4a should be strongly considered for inclusion in future measures. However, it should also be noted that previous studies have shown the PRQ (which was the source DoTS questions 4a and 4b [ever had, currently have tics]) had low sensitivity (<60% for parents or teachers) and moderate specificity (74% for teachers and 92% for parents) based on a single informant (Cubo et al., 2011). The Cubo et al. (2011) study included a school-based population of approximately 1,000 elementary and middle school students and conducted assessments of tic disorders among 334, of which 179 had possible tics. Although the sensitivity analyses presented here are encouraging for the performance of the PRQ in general population settings, other studies have found that while some measures of tic disorders perform in populations recruited from tic specialty clinics, they may not perform as well in general population settings (Bitsko et al., unpublished data from PLAY-MH; Lewin et al., unpublished data from “Tics as a Marker” study; Mårland et al., 2017). Additional research is needed to determine whether collecting information from multiple reporters may improve performance and whether the performance of this question alongside other questions beyond those included in the present analysis may also contribute information to improve the identification of tics.

Compared to the results for the DoTS, there was more variability in MOVeIT-10 item performance across models. For example, item 1 (movements/sounds over and over) was important across all three main sets of random forest models, but only child report on item 1 was significant in the child report alone logistic regression model; item 1 was not one of the most important items in the sensitivity analyses (across models). While item 7 (sounds over and over, hard to keep from doing) was important and significant by both parent and child report in the combined random forest and logistic regression models, it was not one of the most important items in parent-only or child-only models. Items that were not among the most important (and were not significant) in any of the models were parent report on items 2 (sounds, even I don’t want to), 3 ((movements, even if I don’t want to, with examples), and 8 (sounds/movements, even if I don’t want to), and child report on items 3 and 8; thus, these items could be dropped from a revised measure. Given the differences in item importance and significance by parent, child, or combined report, future measure development should consider developing parent- and child- specific measures that may have unique items. Items 4 (movements/sounds over and over, hard to keep from doing, with examples), 5 (movements over and over, with examples), and 10 movements over and over, hard to keep from doing) could be retained for both parent and/or child report, as well as items 1 (movements/sounds over and over), 6 (movements/sounds over and over, with examples), 7 (sounds over and over, hard to keep from doing) for parent or item 2 (sounds, even if I don’t want to) for child report. In addition, redundancy across items can be considered in choosing items for future measures, as well as consideration of including items that assess both motor and vocal tics. In addition, future studies could explore factors that contribute to differences in reporting by parents and children.

With regard to sensitivity in predicting tics, random forest models outperform or perform comparably to logistic regression models. All random forest models achieved sensitivity equal to or greater than the sensitivity achieved by the corresponding logistic regression model. While the accuracy of logistic regression and random forest models can be adjusted through specification of the cutoff value used to classify the model’s prediction, the adjustment is on a much finer scale for random forest models given the increased relative sophistication of the model. These adjustments to the cutoff value ultimately affect the preponderance of Type I and Type II errors. The collection of predicted probabilities from logistic regression models can have no more unique values than there are unique patterns to the questions. Random forest models, on the other hand, have a greatly increased number of unique predicted probabilities, and that is how the scale is much finer. Both approaches yield a final predicted probability. However, logistic regression yields a single set of predicted probabilities while random forest models (via estimation across random subgroups) yield empirical distributions of probabilities that better reflect all sources of variability in the data. In that way, random forest model predictions reflect machine learning and can yield much better results in terms of reduced Type II errors (i.e., reducing false negatives). Further, note that items within the MOVeIT and DoTS questionnaires were often highly correlated. In logistic regression, multi-collinearity can make coefficients in the model sensitive to small changes and reduce the precision of the estimates, weakening the power of the model. However, it shouldn’t influence the prediction of tic disorder. In the random forest variable importance measure, correlation between items may inflate importance but will likely not lead to incorrectly identifying items as important. Therefore, we can trust that items with positive variable importance are, in fact, improving prediction of tic disorder.

Thus, using random forest and machine learning provides an attractive alternative to logistic modeling that may help to identify the best performing items for identifying tics.

There are several limitations to this study. Our constructed models used data combined across studies that had different gold standards. In addition, a significant portion of study participants were drawn from specialty clinics. Thus, our data could include biases that would have been reduced had we been able to assess data from a random population-based sample. These data do not reflect the demographics of the population. In many of the models, children in the “other/multiple” race category had a lower odds of having a tic disorder compared to non-Hispanic White children. However, this may be due to having fewer children in this group who were recruited from the tic specialty clinics and does not necessarily imply this group has a lower prevalence of tic disorders. A future planned study could avoid these issues as well as incorporating extensions of random forest models that could incorporate varying levels of frailty for subsets of the sample population. Future studies could also examine differences in responses by subgroups of participants, including by age, sex, and race/ethnicity.

Despite these limitations, the data presented here provide information on the best items from two current measures for identifying tics and tic disorders. This information can be incorporated into the development of future measures, with a goal of improving identification of tics and tic disorders in population-based settings. Screening measures with optimized sensitivity and specificity have the potential to improve clinical identification and referral for care if needed, as well as to improve identification of tics in future epidemiological studies.

## Supplementary Material

SUP - Wardrop - Improving Identification of Tic Disorders in Children

## Figures and Tables

**Table 1.** Data source information including gold standard used to determine tic disorder status, beginning sample size, number of observations with known tic disorder status and at least one response to an item on the 10-item motor or vocal inventory of tics (MOVeIT-10) screener or the Description of tic Symptoms (DoTS), summary of demographics, and source population.

Study including location and measures (Study Years)	Gold Standard	Sample Size	Observations with Known Tic Disorder Status	Demographics						Population
				Sex – Female (%)	Race – Non-Hispanic White (%)	Race – Non-Hispanic Black (%)	Race – Other (%)	Race – Missing (%)	Age Range, years (Median)	
UF Jacksonville PLAY-MH DoTS (2016–2018)^a^	KSADS-PL^b^	100	93 (92 parent; 92 self)	40 (43.0%)	46 (49.5%)	28 (30.1%)	19 (20.4%)	0 (0.0%)	8–20 (12)	Population-based epidemiologic study
USF^c^ MOVeIT development (2015)	YGTSS^d^	42	37 (35 parent; 26 self)	15 (40.5%)	9 (24.3%)	16 (43.2%)	12 (32.4%)	0 (0.0%)	4–17 (10)	Tic disorder specialty clinic and general pediatric clinic
UR^e^ Validation Study: MOVeIT & DoTS (2016–2017)	Expert Determination	100	100 (99 DoTS (98 parent; 68 self); 100 MOVeIT (100 parent; 67 self)	37 (37.0%)	87 (87.0%)	2 (2.0%)	11 (11.0%)	0 (0.0%)	6–17 (11)	Tic disorder specialty clinic and community controls
UR DBP^f^ Study MOVeIT (2018)	Expert Determination	266	199 (199 parent)	46 (22.8%)	163 (80.7%)	16 (7.9%)	13 (6.4%)	7 (3.5%)	2–16 (7)	Developmental and behavioral pediatrics clinic
USF Tics as a Marker MOVeIT-10 (2019–2021) &	YGTSS, K-SADS-PL	799	776 (292 DoTS (289 parent; 143 self); 486 MOVeIT-10 (484 parent; 230 self)	362 (46.6%)	232 (29.9%)	273 (35.2%)	270 (34.8%) (178	1 (0.1%)	4–17 (9)	Primary care pediatric clinic
USF Tics as a Marker DoTS (2019–2021)							Hispanic (22.9%)			

a UF: University of Florida College of Medicine in Jacksonville; PLAY-MH: Project to Learn about Youth Mental Health.

b Developmental K-SADS-PL: Schedule for Affective Disorder and Schizophrenia for School-Age Children-Present and Lifetime.

c USF: University of South Florida.

d YGTSS: Yale Global Tic Severity Scale.

e UR: University of Rochester Medical Center.

f DBP: Developmental-Behavioral Pediatrics clinic.

**Table 2.** Prediction results for description of tic symptoms (DoTS) models.

Measure	Logistic Regression Full Model	Logistic Regression Full Model adjusted for demographics	Logistic Regression Parent Model	Logistic Regression Parent Model adjusted for demographics	Logistic Regression Child Model	Logistic Regression Child Model adjusted for demographics
Cutoff	0.45	0.48	0.41	0.28	0.40	0.34
Sensitivity (%)	90.9	90.9	85.0	85.0	93.8	93.8
Specificity (%)	95.0	95.0	94.9	94.9	87.2	87.2
Measure	Random Forest Full Model	Random Forest Full Model adjusted for demographics	Random Forest Parent Model	Random Forest Parent Model adjusted for demographics	Random Forest Child Model	Random Forest Child Model adjusted for demographics
Cutoff	0.50	0.50	0.42	0.50	0.23	0.35
Sensitivity (%)	90.9	90.9	85.0	85.0	93.8	93.8
Specificity (%)	92.5	92.5	93.6	93.6	78.7	83.0

Sensitivity and specificity reported at the selected cut off value across 3 studies. Data were included from University of Florida Jacksonville Project to Learn about Youth Mental Health, University of Rochester validation study, and University of South Florida Tics as a Marker study. Demographic variables include sex (male/female), race (non-Hispanic White, non-Hispanic Black, and other or multiples races/ethnicities), and age. Full Models include both child and parent responses to DoTS questionnaires, Parent Models include parent responses to DoTS questionnaires, and Child Models include child responses to DoTS questionnaires.

**Table 3.** Prediction results for the 10-item motor or vocal inventory of tics (MOVeIT-10) models.

Measure	Logistic Regression Full Model	Logistic Regression Full Model adjusted for demographics	Logistic Regression Parent Model	Logistic Regression Parent Model adjusted for demographics	Logistic Regression Child Model	Logistic Regression Child Model adjusted for demographics
Cutoff	0.03	0.04	0.21	0.12	0.05	0.08
Sensitivity (%)	83.3	76.7	78.4	80.4	92.3	84.6
Specificity (%)	72.1	78.7	83.7	74.3	43.5	56.5
Measure	Random Forest Full Model	Random Forest Full Model adjusted for demographics	Random Forest Parent Model	Random Forest Parent Model adjusted for demographics	Random Forest Child Model	Random Forest Child Model adjusted for demographics
Cutoff	0.19	0.02	0.05	0.10	0.03	0.04
Sensitivity (%)	83.3	96.7	84.3	84.3	92.3	96.2
Specificity (%)	88.5	50.8	70.5	71.1	59.4	43.5

Sensitivity and specificity reported at the selected cut off value across 4 studies. Data were included from the University of South Florida MOVeIT development study, University of Rochester validation study, University of Rochester Developmental & Behavioral Pediatrics clinic study, and University of South Florida Tics as a Marker study. Demographic variables include sex (male/female), race (non-Hispanic White, non-Hispanic Black, and other or multiples races/ethnicities), and age. Full models include both child and parent responses to MOVeIT-10 questionnaires, parent models include parent responses to MOVeIT-10 questionnaires, and child models include child responses to MOVeIT-10 questionnaires.

**Table 4.** Random Forest Variable Importance^a^ Results for 10 item motor or vocal inventory of tics (MOVeIT-10) models.

Item	MOVeIT-10	MOVeIT-10 adjusted for demographics	MOVeIT-10 Parent	MOVeIT-10 Parent adjusted for demographics	MOVeIT-10 Child	MOVeIT-10 Child adjusted for demographics
Sex		2.571		−0.332		12.814
Race		8.04		12.79		13.435
Age		−0.517		18.202		0.159
MOVeIT Parent 1	20.186	17.834	2.901	16.614		
MOVeIT Parent 2	3.627	2.205	5.376	7.433		
MOVeIT Parent 3	5.67	5.133	5.085	9.865		
MOVeIT Parent 4	14.083	14.612	19.379	18.816		
MOVeIT Parent 5	15.567	15.301	30.236	27.224		
MOVeIT Parent 6	17.215	15.53	15.16	19.081		
MOVeIT Parent 7	12.827	13.026	1.482	4.779		
MOVeIT Parent 8	6.359	5.361	4.692	9.102		
MOVeIT Parent 9	10.013	9.455	2.877	5.88		
MOVeIT Parent 10	8.222	8.556	15.725	16.107		
MOVeIT Self 1	9.343	9.033			14.391	18.184
MOVeIT Self 2	1.547	4.968			12.338	14.953
MOVeIT Self 3	−1.975	0.591			0.99	6.323
MOVeIT Self 4	7.363	8.317			11.389	16.822
MOVeIT Self 5	1.348	0.751			9.209	8.734
MOVeIT Self 6	5.018	5.492			7.553	14.282
MOVeIT Self 7	4.377	1.876			10.16	11.206
MOVeIT Self 8	1.216	1.297			6.211	4.531
MOVeIT Self 9	22.069	17.18			10.985	14.887
MOVeIT Self 10	8.414	9.009			11.599	11.387

All responses to questionnaire items are treated as categorical. Data were included from the University of South Florida MOVeIT development study, University of Rochester validation study, University of Rochester Developmental & Behavioral Pediatrics clinic study, and University of South Florida Tics as a Marker study.

a Variable importance is measured by the change in accuracy when the variable’s out-of-bag (OOB) values are permuted. Negative variable importance indicates that accuracy improves when OOB values of a variable are permuted.

**Table 5.** Odds ratios with 95% confidence intervals for 10 item motor or vocal inventory of tics (MOVeIT-10) models.

	MOVe IT		MOVe IT adjusted for demographics		MOVe IT Parent		MOVe IT Parent adjusted for demographics		MOVe IT Child		MOVe IT Child adjusted for demographics	
Item	OR^a^ (95% CI^b^)	p-value	OR (95% CI)	p-value	OR (95% CI)	p-value	OR (95% CI)	p-value	OR (95% CI)	p-value	OR (95% CI)	p-value
Sex (female)			0.24* (0.05, 1.10)	.07*			0.68 (0.38, 1.23)	.20			0.19*** (0.07, 0.53)	.00***
Race – Non-Hispanic Black			0.22* (0.04, 1.33)	.10*			0.72 (0.38, 1.40)	.33			0.40* (0.14, 1.18)	.10*
Race – Other/Multiple			0.24 (0.04, 1.47)	.12			0.31*** (0.13, 0.72)	.01***			0.36* (0.11, 1.17)	.09*
Age			0.96 (0.72, 1.27)	.75			1.07* (0.99, 1.15)	.05*			1.05 (0.87, 1.26)	.61
MOVeIT Parent 1	0.76 (0.16, 3.74)	.74	1.04 (0.20, 5.55)	.96	1.49 (0.84, 2.64)	.17	1.42 (0.79, 2.58)	.24				
MOVeIT Parent 2	0.47 (0.09, 2.60)	.39	0.18 (0.02, 1.52)	.12	1.03 (0.59, 1.82)	.91	1.14 (0.63, 2.08)	.67				
MOVeIT Parent 3	1.10 (0.24, 4.93)	.90	0.89 (0.19, 4.20)	.89	0.98 (0.54, 1.77)	.93	1.04 (0.57, 1.91)	.90				
MOVeIT Parent 4	2.14 (0.42, 11.03)	.36	2.92 (0.54, 15.69)	.21	1.36 (0.68, 2.72)	.39	1.33 (0.66, 2.67)	.42				
MOVeIT Parent 5	2.82 (0.58, 13.72)	.20	2.79 (0.53, 14.61)	.22	2.40*** (1.28, 4.51)	.01***	2.35*** (1.23, 4.49)	.01***				
MOVeIT Parent 6	0.92 (0.17, 4.81)	.92	0.67 (0.13, 3.52)	.63	1.82 (0.86, 3.85)	.12	1.66 (0.77, 3.61)	.20				
MOVeIT Parent 7	8.92*** (2.18, 36.50)	<.01***	11.91*** (2.34, 60.54)	<.01***	1.22 (0.63, 2.36)	.56	1.09 (0.54, 2.17)	.82				
MOVeIT Parent 8	0.54 (0.19, 2.48)	.43	0.90 (0.17, 4.84)	.90	1.51 (0.79, 2.88)	.21	1.50 (0.77, 2.91)	.24				
MOVeIT Parent 9	3.95* (0.78, 20.01)	.10*	4.65 (0.71, 30.37)	.11	0.64 (0.36, 1.15)	.14	0.73 (0.39, 1.36)	.32				
MOVeIT Parent 10	1.17 (0.323, 4.16)	.81	0.85 (0.22, 3.19)	.80	1.09 (0.61, 1.95)	.78	0.98 (0.54, 1.79)	.94				
MOVeIT Self 1	2.19 (0.68, 7.08)	.19	2.38 (0.68, 8.30)	.17					2.98*** (1.35, 6.56)	.01***	2.51** (1.11, 5.67)	.03**
MOVeIT Self 2	0.60 (0.19, 1.94)	.39	0.44 (0.11, 1.81)	.26					1.96 (0.83, 4.63)	.13	1.96 (0.73, 5.28)	.18
MOVeIT Self 3	0.94 (0.32, 2.72)	.91	0.95 (0.29, 3.15)	.94					0.93 (0.45, 1.91)	.84	1.05 (0.47, 2.38)	.90
MOVeIT Self 4	2.09 (0.57, 7.65)	.27	1.60 (0.34, 7.55)	.55					1.40 (0.64, 3.05)	.40	1.25 (0.52, 2.97)	.62
MOVeIT Self 5	0.67 (0.21, 2.12)	.49	0.78 (0.22, 2.76)	.70					1.37 (0.66, 2.87)	.40	1.23 (0.56, 2.71)	.61
MOVeIT Self 6	1.35 (0.41, 4.47)	.63	0.92 (0.24, 3.55)	.90					1.52 (0.68, 3.41)	.31	1.27 (0.49, 3.31)	.62
MOVeIT Self 7	0.23** (0.06, 0.84)	.03**	0.22** (0.05, 0.94)	.04**					0.74 (0.29, 1.89)	.53	0.94 (0.34, 2.59)	.90
MOVeIT Self 8	0.50 (0.16, 1.54)	.22	0.55 (0.16, 1.90)	.35					0.39** (0.17, 0.90)	.03**	0.42* (0.17, 1.06)	.07*
MOVeIT Self 9	3.78*** (1.41, 10.18)	.01***	4.63*** (1.55, 13.87)	.01***					1.74 (0.76, 4.00)	.19	1.72 (0.66, 4.52)	.27
MOVeIT Self 10	3.03** (1.16, 7.90)	.02**	4.73*** (1.54, 14.55)	.01***					1.86 (0.84, 4.13)	.13	2.39* (0.97, 5.90)	.06*

Non-dichotomous responses to questionnaire items are treated as continuous. Data were included from the University of South Florida MOVeIT development study, University of Rochester validation study, University of Rochester Developmental & Behavioral Pediatrics clinic study, and University of South Florida Tics as a Marker study.

a CI: 95% confidence interval. Note that 95% confidence intervals correspond to p-values < .05.

b OR: odds ratio.

* *p*-value < .1.

** *p*-value < .05,

*** *p*-value ≤ .01.

**Table 6.** Random forest variable importance^a^ results for description of tic symptoms (DoTS) models.

Item	DoTS	DoTS adjusted for demographics	DoTS Parent	DoTS Parent adjusted for demographics	DoTS Child	DoTS Child adjusted for demographics
Sex		−1.05		−0.68		3.92
Race		4.95		8.28		12.35
Age		5.91		8.13		1.03
DoTS Parent 1a	22.09	23.61	11.04	15.80		
DoTS Parent 1b	8.81	8.89	12.82	11.90		
DoTS Parent 1c	22.57	22.35	22.02	23.32		
DoTS Parent 1d	8.18	8.35	9.27	9.30		
DoTS Parent 1e	−4.24	−2.26	0.20	−1.04		
DoTS Parent 1f	0.85	1.23	8.69	9.48		
DoTS Parent 2	10.07	10.42	9.45	12.93		
DoTS Parent 3	6.35	6.14	5.65	6.24		
DoTS Parent 4a	18.25	17.29	27.10	32.00		
DoTS Parent 4b	9.83	9.80	19.86	21.70		
DoTS Self 1a	2.09	0.68			4.75	4.01
DoTS Self 1b	4.51	1.08			−0.06	−0.07
DoTS Self 1c	7.43	4.30			8.03	9.93
DoTS Self 1d	5.34	5.11			10.64	5.50
DoTS Self 1e	8.32	8.29			1.22	1.41
DoTS Self 1f	0.48	0.67			−1.09	−0.55
DoTS Self 2	−2.12	2.57			0.30	0.68
DoTS Self 3	3.70	2.94			−1.79	−0.38
DoTS Self 4a	14.41	13.04			39.63	30.43
DoTS Self 4b	4.50	5.83			17.62	20.99

All responses to questionnaire items are treated as categorical. Data were included from University of Florida Jacksonville Project to Learn about Youth Mental Health, University of Rochester validation study, and University of South Florida Tics as a Marker study.

a Variable importance is measured by the change in accuracy when the variable’s out-of-bag (OOB) values are permuted. Negative variable importance indicates that accuracy improves when OOB values of a variable are permuted.

**Table 7.** Odds ratios with 95% confidence intervals for description of tic symptoms (DoTS) models.

Item	DoTS		DoTS adjusted for demographics		DoTS Parent		DoTS Parent adjusted for demographics		DoTS Child		DoTS Child adjusted for demographics	
	OR^a^ (95% CI^b^)	p-value	OR (95% CI)	p-value	OR (95% CI)	p-value	OR (95% CI)	p-value	OR (95% CI)	p-value	OR (95% CI)	p-value
Sex (female)			0.88 (0.31, 2.45)	.80			0.84 (0.35, 2.03)	.70			0.40** (0.16, 0.99)	.05**
Race – Non-Hispanic Black			0.39 (0.13, 1.22)	.11			0.66 (0.24, 1.79)	.41			0.40* (0.15, 1.09)	.07*
Race – Other/Multiple			0.19** (0.05, 0.78)	.02**			0.38* (0.13, 1.09)	.07*			0.32* (0.11, 1.00)	.05*
Age			1.05 (0.88, 1.25)	.61			1.08 (0.97, 1.20)	.16			1.03 (0.88, 1.21)	.69
DoTS Parent 1a	2.28* (0.97, 5.34)	0.06*	2.83** (1.12, 7.18)	.03**	1.75* (0.94, 3.26)	0.08*	1.75* (0.91, 3.34)	.09*				
DoTS Parent 1b	0.81 (0.15, 4.49)	0.81	0.88 (0.18, 4.41)	.88	0.96 (0.35, 2.60)	0.93	0.83 (0.31, 2.20)	.71				
DoTS Parent 1c	5.15* (0.84, 31.45)	0.08*	3.20 (0.53, 19.23)	.20	2.51* (0.95, 6.64)	0.06*	2.28 (0.85, 6.07)	.10				
DoTS Parent 1d	1.15 (0.39, 3.38)	0.80	1.35 (0.42, 4.29)	.62	1.17 (0.64, 2.16)	0.61	1.25 (0.66, 2.35)	.49				
DoTS Parent 1e	0.64 (0.21, 1.92)	0.43	0.78 (0.25, 2.40)	.66	0.43** (0.19, 0.98)	0.04**	0.48* (0.21, 1.07)	.07*				
DoTS Parent 1f	0.86 (0.39, 1.87)	0.70	0.82 (0.35, 1.90)	.64	1.13 (0.66, 1.95)	0.66	1.15 (0.66, 2.00)	.63				
DoTS Parent 2	0.86 (0.11, 6.72)	0.89	1.55 (0.16, 15.19)	.71	2.20 (0.80, 6.05)	0.13	2.67* (0.93, 7.70)	.07*				
DoTS Parent 3	1.10 (0.22, 5.49)	0.91	0.82 (0.13, 5.26)	.83	1.92 (0.68, 5.43)	0.22	1.92 (0.66, 5.60)	.24				
DoTS Parent 4a	6.85** (1.36, 34.49)	0.02**	4.68* (0.78, 28.07)	.09*	5.30** (1.25, 22.39)	0.02***	4.66** (1.11, 19.50)	.04**				
DoTS Parent 4b	1.19 (0.12, 11.65)	0.88	1.44 (0.10, 20.13)	.79	2.01 (0.40, 10.18)	0.4	1.90 (0.38, 9.31)	.43				
DoTS Self 1a	0.85 (0.37, 1.92)	0.69	0.76 (0.31, 1.88)	.56					1.56 (0.86, 2.85)	.15	1.35 (0.71, 2.57)	.36
DoTS Self 1b	0.46 (0.18, 1.17)	0.10	0.34** (0.12, 0.99)	.05**					0.64 (0.32, 1.29)	.21	0.64 (0.30, 1.38)	.25
DoTS Self 1c	1.21 (0.53, 2.75)	0.66	1.53 (0.63, 3.72)	.35					1.77* (0.92, 3.42)	.09*	1.82* (0.92, 3.57)	.08*
DoTS Self 1d	1.30 (0.68, 2.46)	0.43	1.41 (0.69, 2.89)	.35					1.97** (1.13, 3.41)	.02**	1.67 (0.87, 3.21)	.12
DoTS Self 1e	1.06 (0.58, 1.94)	0.85	1.23 (0.66, 2.30)	.52					0.55** (0.32, 0.95)	.03**	0.64 (0.36, 1.12)	.12
DoTS Self 1f	0.88 (0.49, 1.60)	0.68	0.85 (0.44, 1.65)	.64					0.62** (0.38, 0.99)	.05**	0.75 (0.44, 1.28)	.29
DoTS Self 2	2.188 (0.78, 6.11)	0.14	2.02 (0.70, 5.84)	.20					1.61 (0.68, 3.84)	.28	1.55 (0.62, 3.85)	.35
DoTS Self 3	1.49 (0.50, 4.42)	0.47	1.60 (0.50, 5.13)	.43					1.48 (0.59, 3.70)	.41	1.78 (0.65, 4.83)	.26
DoTS Self 4a	5.67*** (1.64, 19.61)	0.01***	8.55*** (2.13, 34.37)	<.01***					10.09*** (3.54, 28.73)	<.01***	9.84*** (3.11, 31.14)	<.01***
DoTS Self 4b	0.49 (0.12, 2.03)	0.32	0.35 (0.08, 1.62)	.18					1.16 (0.39, 3.54)	.79	0.81 (0.25, 2.64)	.73

Non-dichotomous responses to questionnaire items are treated as continuous. Data were included from University of Florida Jacksonville Project to Learn about Youth Mental Health, University of Rochester validation study, and University of South Florida Tics as a Marker study.

a CI: 95% confidence interval. Note that 95% confidence intervals correspond to p-values <.05.

b OR: odds ratio.

* *p*-value < .1.

** *p*-value < .05,

*** *p*-value ≤ .01.
