# Supplementary material for: Improving Identification of Tic Disorders in Children
Source: Evid Based Pract Child Adolesc Ment Health. Author manuscript; Available in PMC 2025 Mar 21. (PMC11328645; doi:10.1080/23794925.2024.2324775)
Supplement: SUP - Wardrop - Improving Identification of Tic Disorders in Children [file NIHMS1986304-supplement-SUP_-_Wardrop_-_Improving_Identification_of_Tic_Disorders_in_Children.docx]

**Supplemental Table 1**

Random Forest Variable Importance^a^ Results for Description of Tic Symptoms (DoTS) Models in the Sensitivity Analysis

| **Item** | **DoTS** | **DoTS adjusted for demographics** | **DoTS Parent** | **DoTS Parent adjusted for demographics** | **DoTS Child** | **DoTS Child adjusted for demographics** |
| --- | --- | --- | --- | --- | --- | --- |
| Sex |  | -2.10 |  | 3.65 |  | -1.11 |
| Race |  | -0.79 |  | -4.06 |  | -0.09 |
| Age |  | 5.79 |  | 10.56 |  | 2.81 |
| DoTS Parent 1a | 13.04 | 15.88 | 8.55 | 18.15 |  |  |
| DoTS Parent 1b | 2.50 | 1.62 | 5.25 | 0.04 |  |  |
| DoTS Parent 1c | 9.03 | 8.46 | 1.26 | 1.52 |  |  |
| DoTS Parent 1d | -0.91 | -2.54 | -3.46 | -8.32 |  |  |
| DoTS Parent 1e | -3.52 | -4.07 | 1.45 | -1.97 |  |  |
| DoTS Parent 1f | 2.29 | 1.83 | 0.15 | -9.38 |  |  |
| DoTS Parent 2 | 2.52 | 4.24 | 1.13 | 0.94 |  |  |
| DoTS Parent 3 | 1.38 | 0.40 | 1.77 | -3.87 |  |  |
| DoTS Parent 4a | 5.73 | 5.74 | 6.98 | 10.50 |  |  |
| DoTS Parent 4b | 0.92 | 2.21 | 3.71 | 6.68 |  |  |
| DoTS Self 1a | 6.54 | 4.06 |  |  | -2.20 | -3.61 |
| DoTS Self 1b | 2.69 | -0.90 |  |  | -0.46 | -0.01 |
| DoTS Self 1c | 8.73 | 7.07 |  |  | 4.73 | 4.92 |
| DoTS Self 1d | 4.34 | 1.54 |  |  | -0.49 | -0.20 |
| DoTS Self 1e | 4.43 | 4.83 |  |  | 2.40 | 2.25 |
| DoTS Self 1f | 3.85 | 3.00 |  |  | -1.34 | -0.94 |
| DoTS Self 2 | -0.39 | 1.39 |  |  | -1.88 | -0.83 |
| DoTS Self 3 | -0.41 | 1.33 |  |  | -1.19 | -0.23 |
| DoTS Self 4a | 4.84 | 5.51 |  |  | 5.13 | 5.10 |
| DoTS Self 4b | 0.92 | 0.27 |  |  | 1.29 | 2.41 |

*Note.* All responses to questionnaire items are treated as categorical. Data were included from University of Florida Jacksonville Project to Learn about Youth Mental Health, and University of South Florida Tics as a Marker study.

^a^ Variable importance is measured by the change in accuracy when the variable’s out-of-bag (OOB) values are permuted. Negative variable importance indicates that accuracy improves when OOB values of a variable are permuted.

**Supplemental Table 2**

Odds Ratios with 95% Confidence Intervals for Description of Tic Symptoms (DoTS) Models in Sensitivity Analysis

| **Item** | **DoTS** | | **DoTS adjusted for demographics** | | **DoTS Parent** | | **DoTS Parent adjusted for demographics** | | **DoTS Self** | | **DoTS Self adjusted for demographics** | |
| --- | --- | --- | --- | --- | --- | --- | --- | --- | --- | --- | --- | --- |
|  | **OR^a^ (95% CI^b^)** | **p-value** | **OR (95% CI)** | **p-value** | **OR (95% CI)** | **p-value** | **OR (95% CI)** | **p-value** | **OR (95% CI)** | **p-value** |  | **p-value** |
| Sex (female) |  |  | 1.1 (0.37, 3.27) | 0.86 |  |  | 0.60 (0.26, 1.39) | 0.23 |  |  | 0.46* (0.18, 1.15) | 0.10* |
| Race - Non-Hispanic Black |  |  | 0.6 (0.2, 1.8) | 0.36 |  |  | 0.63 (0.25, 1.59) | 0.32 |  |  | 0.58 (0.22, 1.54) | 0.27 |
| Race - Other/Multiple |  |  | 0.15** (0.03, 0.69) | 0.02** |  |  | 0.46 (0.17, 1.24) | 0.12 |  |  | 0.26** (0.08, 0.84) | 0.02** |
| Age |  |  | 0.94 (0.8, 1.1) | 0.43 |  |  | 1.13** (1.02, 1.24) | 0.02** |  |  | 0.87 (0.74, 1.03) | 0.10 |
| DoTS Parent 1a | 2.06* (0.89, 4.76) | 0.09* | 2.22* (0.91, 5.41) | 0.08* | 2.82*** (1.33, 6) | 0.01*** | 3.06*** (1.4, 6.69) | 0.01*** |  |  |  |  |
| DoTS Parent 1b | 1.18 (0.25, 5.6) | 0.83 | 1.46 (0.3, 7.03) | 0.63 | 1.02 (0.33, 3.15) | 0.98 | 0.78 (0.25, 2.40) | 0.66 |  |  |  |  |
| DoTS Parent 1c | 3.55 (0.75, 16.75) | 0.11 | 2.72 (0.57, 13.08) | 0.21 | 1.18 (0.33, 4.26) | 0.8 | 1.45 (0.39, 5.40) | 0.58 |  |  |  |  |
| DoTS Parent 1d | 0.82 (0.15, 4.64) | 0.82 | 0.84 (0.13, 5.24) | 0.85 | 0.51 (0.21, 1.23) | 0.14 | 0.60 (0.25, 1.45) | 0.26 |  |  |  |  |
| DoTS Parent 1e | 0.61 (0.18, 2.09) | 0.43 | 0.61 (0.16, 2.31) | 0.47 | 0.47* (0.20, 1.1) | 0.08* | 0.54 (0.23, 1.27) | 0.16 |  |  |  |  |
| DoTS Parent 1f | 0.87 (0.39, 1.93) | 0.73 | 0.76 (0.32, 1.8) | 0.53 | 1.24 (0.73, 2.11) | 0.42 | 1.20 (0.69, 2.09) | 0.52 |  |  |  |  |
| DoTS Parent 2 | 1.29 (0.23, 7.30) | 0.77 | 3.03 (0.41, 22.2) | 0.28 | 2.30 (0.79, 6.70) | 0.13 | 2.85* (0.91, 8.96) | 0.07* |  |  |  |  |
| DoTS Parent 3 | 0.65 (0.10, 4.07) | 0.64 | 0.55 (0.07, 4.13) | 0.56 | 1.14 (0.37, 3.54) | 0.82 | 0.92 (0.28, 3.01) | 0.89 |  |  |  |  |
| DoTS Parent 4a | 5.17** (1.09, 24.61) | 0.04** | 4.61* (0.81, 26.26) | 0.09* | 2.67 (0.69, 10.36) | 0.16 | 2.36 (0.60, 9.37) | 0.22 |  |  |  |  |
| DoTS Parent 4b | 0.84 (0.08, 8.88) | 0.89 | 1.27 (0.09, 17.13) | 0.86 | 1.10 (0.20, 6.23) | 0.91 | 1.16 (0.21, 6.52) | 0.87 |  |  |  |  |
| DoTS Self 1a | 1.05 (0.38, 2.87) | 0.92 | 0.98 (0.31, 3.11) | 0.97 |  |  |  |  | 0.89 (0.36, 2.19) | 0.8 | 0.74 (0.26, 2.09) | 0.57 |
| DoTS Self 1b | 0.64 (0.25, 1.65) | 0.35 | 0.61 (0.23, 1.62) | 0.32 |  |  |  |  | 0.46 (0.18, 1.17) | 0.10 | 0.40* (0.15, 1.04) | 0.06* |
| DoTS Self 1c | 1.62 (0.69, 3.80) | 0.27 | 1.84 (0.74, 4.56) | 0.19 |  |  |  |  | 1.82 (0.88, 3.78) | 0.11 | 2.38** (1.12, 5.07) | 0.02** |
| DoTS Self 1d | 1.21 (0.60, 2.45) | 0.59 | 1.11 (0.51, 2.41) | 0.8 |  |  |  |  | 1.3 (0.73, 2.29) | 0.37 | 0.91 (0.46, 1.79) | 0.78 |
| DoTS Self 1e | 1.22 (0.67, 2.20) | 0.52 | 1.28 (0.69, 2.37) | 0.43 |  |  |  |  | 1.08 (0.63, 1.84) | 0.79 | 1.13 (0.66, 1.95) | 0.65 |
| DoTS Self 1f | 0.71 (0.37, 1.34) | 0.29 | 0.67 (0.33, 1.33) | 0.25 |  |  |  |  | 0.95 (0.55, 1.64) | 0.86 | 1.10 (0.62, 1.95) | 0.74 |
| DoTS Self 2 | 3.90*** (1.30, 11.65) | 0.01 | 3.36 (1.1, 10.31) | 0.2 |  |  |  |  | 1.7 (0.65, 4.47) | 0.28 | 1.61 (0.59, 4.38) | 0.35 |
| DoTS Self 3 | 0.76 (0.22, 2.65) | 0.66 | 0.58 (0.14, 2.34) | 0.45 |  |  |  |  | 1.16 (0.42, 3.23) | 0.78 | 1.08 (0.36, 3.22) | 0.89 |
| DoTS Self 4a | 3.05* (0.90, 10.29) | 0.07* | 4.23** (1.11, 16.16) | 0.03** |  |  |  |  | 5.01*** (1.59, 15.87) | 0.01*** | 5.21*** (1.5, 18.16) | 0.01*** |
| DoTS Self 4b | 0.25 (0.05, 1.31) | 0.10 | 0.21* (0.04, 1.15) | 0.07* |  |  |  |  | 0.29* (0.07, 1.21) | 0.09* | 0.30 (0.07, 1.28) | 0.10 |

*Note.* Non-dichotomous responses to questionnaire items are treated as continuous. Data were included from University of Florida Jacksonville Project to Learn about Youth Mental Health, and University of South Florida Tics as a Marker study.

^a^ CI: 95% confidence interval. Note that 95% confidence intervals correspond to p-values < 0.05

^b^ OR: odds ratio

* p-value < 0.1. ** p-value < 0.05, *** p-value ≤ 0.01.

**Supplemental Table 3**

*Random Forest Variable Importance^a^ Results for the 10-item Motor or Vocal Inventory of Tics (MOVeIT-10) Models in Sensitivity Analysis*

| **Item** | **MOVeIT-10** | **MOVeIT-10 adjusted for demographics** | **MOVeIT-10 Parent** | **MOVeIT-10 adjusted for demographics** | **MOVeIT-10 Child** | **MOVeIT-10 Child adjusted for demographics** |
| --- | --- | --- | --- | --- | --- | --- |
| Sex |  | -3.51 |  | -1.43 |  | 1.10 |
| Race |  | -2.30 |  | -1.14 |  | -2.78 |
| Age |  | 2.30 |  | 3.12 |  | -1.51 |
| MOVeIT Parent 1 | 4.92 | 7.52 | 1.74 | 3.66 |  |  |
| MOVeIT Parent 2 | -0.29 | -2.06 | 0.94 | 1.01 |  |  |
| MOVeIT Parent 3 | 1.21 | -0.56 | 1.96 | 1.67 |  |  |
| MOVeIT Parent 4 | 6.75 | 8.32 | 9.92 | 9.16 |  |  |
| MOVeIT Parent 5 | 4.74 | 4.62 | 10.60 | 11.88 |  |  |
| MOVeIT Parent 6 | 5.39 | 5.23 | 10.78 | 10.02 |  |  |
| MOVeIT Parent 7 | 5.11 | 7.69 | 5.20 | 4.75 |  |  |
| MOVeIT Parent 8 | 1.36 | 1.10 | -0.39 | 0.43 |  |  |
| MOVeIT Parent 9 | 2.25 | 0.58 | 2.15 | 2.09 |  |  |
| MOVeIT Parent 10 | 3.05 | 2.27 | 5.61 | 5.30 |  |  |
| MOVeIT Self 1 | 0.85 | 2.00 |  |  | 0.32 | 0.57 |
| MOVeIT Self 2 | 2.10 | 2.16 |  |  | 7.78 | 4.80 |
| MOVeIT Self 3 | 1.34 | 5.44 |  |  | -1.38 | 0.90 |
| MOVeIT Self 4 | 3.05 | 3.04 |  |  | 0.39 | 2.73 |
| MOVeIT Self 5 | 3.45 | 4.79 |  |  | 2.12 | -0.86 |
| MOVeIT Self 6 | 0.42 | 2.25 |  |  | -5.49 | -1.92 |
| MOVeIT Self 7 | 0.62 | 1.68 |  |  | -1.33 | -2.18 |
| MOVeIT Self 8 | -0.18 | 1.50 |  |  | -1.53 | -0.34 |
| MOVeIT Self 9 | 0.77 | 0.82 |  |  | -1.49 | 0.09 |
| MOVeIT Self 10 | 3.54 | 2.75 |  |  | 5.38 | 3.27 |

*Note.* All responses to questionnaire items are treated as categorical. Data were included from the University of South Florida Tics as a Marker study.

^a^ Variable importance is measured by the change in accuracy when the variable’s out-of-bag (OOB) values are permuted. Negative variable importance indicates that accuracy improves when OOB values of a variable are permuted.

**Supplemental Table 4**

Odds Ratios with 95% Confidence Intervals for 10 item Motor or Vocal Inventory of Tics (MOVeIT-10) Models in Sensitivity Analysis

| **Item** | **MOVeIT-10** | | **MOVeIT-10 adjusted for demographics** | | **MOVeIT-10 Parent** | | **MOVeIT-10 Parent adjusted for demographics** | | **MOVeIT-10 Self** | | **MOVeIT-10 Self adjusted for demographics** | |
| --- | --- | --- | --- | --- | --- | --- | --- | --- | --- | --- | --- | --- |
|  | **OR^a^ (95% CI^b^)** | **p-value** | **OR (95% CI)** | **p-value** | **OR (95% CI)** | **p-value** | **OR (95% CI)** | **p-value** | **OR (95% CI)** | **p-value** | **OR (95% CI)** | **p-value** |
| Sex (female) |  |  | 0.49 (0.09, 2.62) | 0.41 |  |  | 0.61 (0.31, 1.19) | 0.15 |  |  | 0.34* (0.09, 1.21) | 0.10* |
| Race - Non-Hispanic Black |  |  | 0.60 (0.10, 3.68) | 0.58 |  |  | 0.85 (0.40, 1.80) | 0.67 |  |  | 0.91 (0.25, 3.38) | 0.89 |
| Race - Other/Multiple |  |  | 0.88 (0.14, 5.30) | 0.89 |  |  | 1.00 (0.50, 2.01) | 0.99 |  |  | 0.69 (0.16, 3.07) | 0.63 |
| Age |  |  | 0.80 (0.53, 1.21) | 0.29 |  |  | 1.05 (0.97, 1.14) | 0.20 |  |  | 1.02 (0.80, 1.30) | 0.87 |
| MOVeIT Parent 1 | 1.56 (0.27, 8.95) | 0.62 | 1.49 (0.21, 10.84) | 0.69 | 1.55 (0.81, 2.96) | 0.19 | 1.49 (0.78, 2.85) | 0.23 |  |  |  |  |
| MOVeIT Parent 2 | 0.54 (0.06, 4.71) | 0.58 | 0.61 (0.06, 5.95) | 0.67 | 0.83 (0.45, 1.52) | 0.54 | 0.89 (0.48, 1.65) | 0.72 |  |  |  |  |
| MOVeIT Parent 3 | 1.55 (0.32, 7.40) | 0.59 | 1.25 (0.24, 6.46) | 0.79 | 1.06 (0.56, 2.00) | 0.86 | 1.08 (0.57, 2.07) | 0.81 |  |  |  |  |
| MOVeIT Parent 4 | 3.39 (0.35, 32.89) | 0.29 | 5.93 (0.41, 85.25) | 0.19 | 1.37 (0.68, 2.74) | 0.37 | 1.40 (0.70, 2.80) | 0.35 |  |  |  |  |
| MOVeIT Parent 5 | 1.49 (0.16, 13.53) | 0.72 | 1.96 (0.19, 20.03) | 0.57 | 1.44 (0.74, 2.79) | 0.28 | 1.41 (0.72, 2.76) | 0.31 |  |  |  |  |
| MOVeIT Parent 6 | 1.70 (0.10, 28.95) | 0.71 | 1.24 (0.06, 24.57) | 0.89 | 2.30** (1.11, 4.77) | 0.03** | 2.13** (1.01, 4.48) | 0.05** |  |  |  |  |
| MOVeIT Parent 7 | 6.20** (1.27, 30.35) | 0.02 | 7.90** (1.28, 48.78) | 0.03** | 0.85 (0.41, 1.75) | 0.66 | 0.80 (0.39, 1.66) | 0.55 |  |  |  |  |
| MOVeIT Parent 8 | 0.65 (0.10, 4.14) | 0.65 | 0.66 (0.09, 5.05) | 0.69 | 0.98 (0.46, 2.07) | 0.95 | 0.97 (0.46, 2.06) | 0.94 |  |  |  |  |
| MOVeIT Parent 9 | 0.25 (0.02, 2.66) | 0.25 | 0.09 (0.00, 2.19) | 0.14 | 0.99 (0.54, 1.79) | 0.97 | 1.05 (0.57, 1.92) | 0.89 |  |  |  |  |
| MOVeIT Parent 10 | 0.53 (0.10, 2.74) | 0.45 | 0.44 (0.08, 2.32) | 0.33 | 1.08 (0.58, 2.01) | 0.8 | 1.07 (0.57, 2) | 0.84 |  |  |  |  |
| MOVeIT Self 1 | 1.11 (0.29, 4.26) | 0.88 | 1.11 (0.26, 4.64) | 0.89 |  |  |  |  | 1.32 (0.46, 3.77) | 0.61 | 1.33 (0.47, 3.72) | 0.59 |
| MOVeIT Self 2 | 1.52 (0.45, 5.19) | 0.50 | 1.93 (0.49, 7.5) | 0.35 |  |  |  |  | 2.58* (0.92, 7.24) | 0.07* | 2.69* (0.95, 7.56) | 0.06* |
| MOVeIT Self 3 | 2.65 (0.81, 8.69) | 0.11 | 3.53* (0.88, 14.1) | 0.07* |  |  |  |  | 0.94 (0.34, 2.58) | 0.91 | 1.01 (0.35, 2.94) | 0.98 |
| MOVeIT Self 4 | 0.54 (0.08, 3.52) | 0.52 | 0.75 (0.10, 5.41) | 0.78 |  |  |  |  | 0.93 (0.30, 2.86) | 0.90 | 0.93 (0.30, 2.93) | 0.90 |
| MOVeIT Self 5 | 1.13 (0.27, 4.69) | 0.87 | 1.02 (0.23, 4.54) | 0.98 |  |  |  |  | 0.75 (0.25, 2.26) | 0.61 | 0.80 (0.26, 2.47) | 0.70 |
| MOVeIT Self 6 | 2.71 (0.55, 13.25) | 0.22 | 1.88 (0.33, 10.75) | 0.48 |  |  |  |  | 2.06 (0.64, 6.64) | 0.23 | 1.57 (0.44, 5.61) | 0.49 |
| MOVeIT Self 7 | 0.41 (0.09, 1.82) | 0.24 | 0.35 (0.07, 1.72) | 0.20 |  |  |  |  | 0.44 (0.14, 1.38) | 0.16 | 0.55 (0.16, 1.87) | 0.34 |
| MOVeIT Self 8 | 0.75 (0.19, 2.99) | 0.68 | 0.67 (0.15, 2.97) | 0.60 |  |  |  |  | 0.78 (0.25, 2.43) | 0.67 | 0.87 (0.27, 2.81) | 0.81 |
| MOVeIT Self 9 | 1.34 (0.38, 4.7) | 0.65 | 1.29 (0.34, 4.87) | 0.71 |  |  |  |  | 1.84 (0.65, 5.26) | 0.25 | 1.72 (0.56, 5.26) | 0.34 |
| MOVeIT Self 10 | 2.35 (0.59, 9.41) | 0.23 | 2.71 (0.65, 11.34) | 0.17 |  |  |  |  | 2.14 (0.81, 5.64) | 0.12 | 2.08 (0.79, 5.47) | 0.14 |

*Note.* Non-dichotomous responses to questionnaire items are treated as continuous. Data were included from the University of South Florida Tics as a Marker study.

^a^ CI: 95% confidence interval. Note that 95% confidence intervals correspond to p-values < 0.05

^b^ OR: odds ratio

* p-value < 0.1. ** p-value < 0.05, *** p-value ≤ 0.01.
